# Distal phalangeal erythema in an infant with biallelic *PDSS1* mutations: Expanding the phenotype of primary Coenzyme Q_10_ deficiency

**DOI:** 10.1002/jmd2.12216

**Published:** 2021-09-15

**Authors:** Marcello Bellusci, Maria Teresa García‐Silva, Ana Martínez de Aragón, Miguel Angel Martín

**Affiliations:** ^1^ Reference Center for Inherited Metabolic Disorders, MetabERN Center “12 de Octubre” University Hospital Madrid Spain; ^2^ Mitochondrial & Neuromuscular Disorders Research Group, Instituto de Investigación Sanitaria “12 de Octubre” (imas12) Madrid Spain; ^3^ Spanish Biomedical Research Networking Center in Rare Diseases (CIBERER) Madrid Spain; ^4^ Pediatric Neuroradiology “12 de Octubre” University Hospital Madrid Spain

**Keywords:** coenzyme Q_10_, cutaneous, phalangeal, erythema, mitochondria, PDSS1

## Abstract

We report a detailed clinical examination in a patient with primary coenzyme Q_10_ deficiency caused by biallelic mutations in the *PDSS1* gene who presented clinical features of mitochondrial encephalopathy associated with pulmonary hypertension, livedo reticularis and particularly, chronic distal phalangeal erythema. Laboratory testing showed elevated plasma lactate and 3‐methyl‐glutaconic and tricarboxylic aciduria. Supplementation with high dose of coenzyme Q_10_ was not effective to control disease progression and the patient died at the age of 3 years old because of a progressive multisystem disorder. Cutaneous involvement in mitochondrial disease is heterogenous, including proliferative, inflammatory, and dystrophic changes among others. The coexistence in our case of phalangeal erythema, livedo reticularis, and pulmonary hypertension suggests microvascular dysfunction as a possible underlying mechanism. This is the first reported patient with *PDSS1* mutations presenting with 3‐methyl‐glutaconic aciduria and distal phalangeal erythema, expanding the phenotype of primary coenzyme Q_10_ deficiency.

We report the detailed clinical examination of an infant with primary coenzyme Q_10_ (CoQ_10_) deficiency, previously published in a case series,^1^ presenting unusual finger findings.

He was born prematurely after in vitro fertilization pregnancy, complicated by twin‐twin transfusion.

At the age of 5 months, he presented failure to thrive, hypotonia, feeding difficulties, bilateral hearing impairment, pulmonary hypertension, livedo reticularis, and distal phalangeal erythema (Figure 1). Laboratory tests showed anemia, lactic acidosis, 3‐methyl‐glutaconic, and tricarboxylic aciduria and decreased CoQ_10_ levels in lymphocytes. At this time, oral ubidecarenone was started at 15 mg/kg/day. Three months later, when biallelic *PDSS1* mutations were identified (NM_014317.3: c.716 T > G (p.Val239Gly) and NM_014317.3: c.1183C > T (p.Arg395*)), ubidecarenone was increased to 30 mg/kg/day.

**FIGURE 1:**
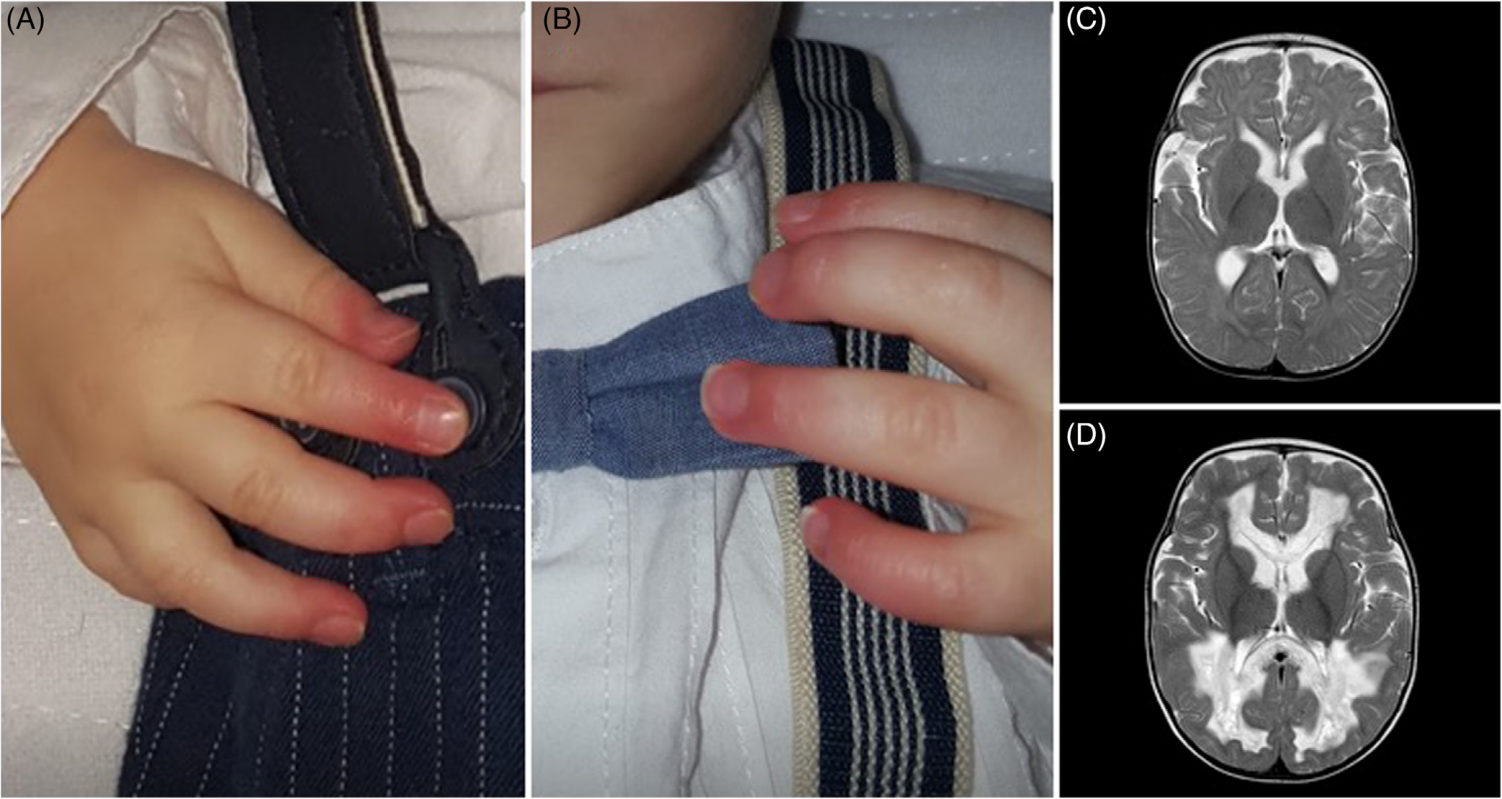
Finger findings and cerebral MRI in the reported patient. A and B, Chronic distal phalangeal erythema, an accompanying sign since the age of 5 months old. C, Axial T2W at the age of 12 months old showing mild cerebral atrophy. D, Axial T2W at the age of 24 months old with appearance of cystic change in white matter. White matter tracts surrounding the lateral ventricles and commissural fibres had signal abnormalities, showing small necrotic or porencephalic cysts. “U” fibres, pyramidal tracts and gray matter were spared

However, he developed a progressive multisystem disorder with severe developmental delay, pyramidal dysfunction, seizures, tremor, optic atrophy and nystagmus, severe visual and hearing impairment, white matter cystic change and chronic heart failure. He died at the age of 3 years old.

Since the age of 5 months, distal phalangeal erythema was an accompanying sign. Cutaneous involvement in mitochondrial disease is heterogenous, including proliferative, inflammatory, and dystrophic changes among others.^2^, ^3^, ^4^ The coexistence in our case of phalangeal erythema, livedo reticularis, and pulmonary hypertension suggests microvascular dysfunction as a possible underlying physiopathogenic mechanism. Symptoms related to microvascular dysfunction and cutaneous involvement have been previously reported in primary CoQ_10_ deficiencies,^5^ (eg, livedo reticularis and pulmonary hypertension in *PDSS1*,^6^ acrocyanosis in *COQ4*,^7^ and lupus‐like symptoms in *CoQ8A* ^8^). In conclusion, our data expand the phenotypic spectrum associated with *PDSS1* variants and primary coenzyme Q_10_ deficiency.

## CONFLICT OF INTEREST

Marcello Bellusci, Maria Teresa García‐Silva, Ana Martínez de Aragón, and Miguel Angel Martín declare that they have no conflict of interest.

## AUTHOR CONTRIBUTION

Marcello Bellusci and Maria Teresa García‐Silva are the clinician who attended the reported patient, planned, wrote and revisited critically the article before the submission. Ana Martínez de Aragón is the pediatric radiologist that realized the reported brain MRI studies, wrote the figure legend and revisited critically the article before the submission. Miguel Angel Martín performed and analyzed laboratory tests, revisited critically the article before the submission.

## INFORMED CONSENT

All procedures followed were in accordance with the ethical standards of the responsible committee on human experimentation (institutional and national) and with the Helsinki Declaration of 1975, as revised in 2000 (5). Informed consent was obtained from all patients for being included in the study, including for the use of pictures.

## ETHICS APPROVAL

No ethics approval is required for case report in our center.
